# COULD WE CARE LESS? FICTIONAL REPRESENTATIONS OF TIME-RESTRICTED IN-HOME-CAREGIVING IN “DIE PFLEGIONÄRIN”

**DOI:** 10.1093/geroni/igad104.0007

**Published:** 2023-12-21

**Authors:** Eva-Maria Trinkaus

**Affiliations:** University of Graz, Graz, Steiermark, Austria

## Abstract

In this narrative film analysis, I look at the representations of care work and its time restrictions in the German TV series “Die Pflegionärin.” In this TV series the lack of adequate time and, thereby, struggle for adequate care in domestic care work becomes visible through ten-minute episodes. Through this representation, both, the perspective of the old patients and their care-needs, as well as the caregiver’s perspective and the pressure they have to perform under becomes visible. In this contribution, I will specifically focus on the representation of the relationship between caregiver and patient, and the possibilities of interaction in a restricted period of time that is captured through the way the fictional representation of care is put on screen. Adding to the pressure that the aspect of time creates, the TV series’ title “Die Pflegionärin“ (transl. “The Carionnaire”) is a linguistic blend of carer and legionnaire, and a war metaphor that highlights the struggle of domestic care even further. By allowing the viewers to follow an in-home-caregiver on her everyday tasks ‘on the front line’ of everyday life, not only the caregiver’s perspective, but also the patients’ needs become visible. Despite the short air time, rather than providing a quick glimpse into the patient’s life, the series provides the viewer with insights into the profession’s tasks and duties through the personal, though fictional, stories of patients and nurse and blends the generic conventions of TV series with real-time in-home caregiving.

